# Mental Health and the COVID-19 Pandemic: Observational Evidence from Malaysia

**DOI:** 10.3390/ijerph20054046

**Published:** 2023-02-24

**Authors:** Eugenie Sin Sing Tan, Shaun Ashley Fung Xian Chin, Manimeyapan S. Palaniappan Sathapan, Astrid Disimond Dewi, Farahnaz Amini, Normina Ahmad Bustami, Pui Yee Tan, Yu Bin Ho, Chung Keat Tan

**Affiliations:** 1 Faculty of Medicine and Health Sciences, UCSI University, Kuala Lumpur 56000, Malaysia; 2 Faculty of Environment, University of Leeds, Leeds LS2 9JT, UK; 3 Faculty of Medicine and Health Sciences, University Putra Malaysia, Serdang 43400, Malaysia

**Keywords:** Coronavirus Disease 2019 (COVID-19), level of knowledge, precautionary behaviors, Depression, Anxiety, and Stress Scales (DASS), World Health Organization Quality of Life—Brief Version (WHOQOL-BREF)

## Abstract

The interplay of physical, social, and economic factors during the pandemic adversely affected the mental health of healthy people and exacerbated pre-existing mental disorders. This study aimed to determine the impact of the COVID-19 pandemic on the mental health of the general population in Malaysia. A cross-sectional study involving 1246 participants was conducted. A validated questionnaire consisting of the level of knowledge and practice of precautionary behaviors, the Depression, Anxiety, and Stress Scales (DASS), and the World Health Organization Quality of Life—Brief Version (WHOQOL-BREF) was used as an instrument to assess the impacts of the COVID-19 pandemic. Results revealed that most participants possessed a high level of knowledge about COVID-19 and practiced wearing face masks daily as a precautionary measure. The average DASS scores were beyond the mild to moderate cut-off point for all three domains. The present study found that prolonged lockdowns had significantly impacted (*p* < 0.05), the mental health of the general population in Malaysia, reducing quality of life during the pandemic. Employment status, financial instability, and low annual incomes appeared to be risk factors (*p* < 0.05) contributing to mental distress, while older age played a protective role (*p* < 0.05). This is the first large-scale study in Malaysia to assess the impacts of the COVID-19 pandemic on the general population.

## 1. Introduction

Global health is threatened by the ongoing outbreak of the respiratory disease named Coronavirus Disease 2019 (COVID-19) [1]. The disease is caused by a single, positive-strand RNA virus known as SARS-CoV-2, which was initially reported in Wuhan, Hubei Province, China [2]. Transmission of COVID-19 occurs mainly through respiratory droplets, and its estimated basic reproduction number (R_0_) ranges from 1.5 to 3.5 [3]. Its relatively high infectivity, long incubation period, long viral shedding period, and steadfast spreading to almost all continents led the World Health Organization to declare a pandemic on 12 March 2020 [2]. As of 8 July 2022, WHO reported more than 550 million confirmed COVID-19 cases, including more than 6 million mortalities [4].

Malaysia is the third-highest country for the number of COVID-19 cases and the fourth-highest country for the number of COVID-19 deaths within the Southeast Asian region [5,6]. The Malaysian government implemented a series of quarantine policies to halt the transmission of COVID-19. In the year 2020, there were four phases of Movement Control Order (MCO) from 18 March to 12 May 2020, two phases of Conditional Movement Control Order (CMCO) from 13 May 2020 to 9 June 2020, and three phases of Recovery Movement Control Order from 10 June 2020 to 31 March 2021 [7,8,9]. In mid-2021, Malaysia declared yet another nationwide Full Movement Control Order (FMCO) from 1 June to 28 June amid a surge of daily COVID-19 cases to 8000 [10].

Pandemics were associated with various psychosocial stressors involving oneself and loved ones. People experienced significant disruptions to their daily routines, such as financial incomes [11], restricted outdoor activities [12], sleep cycles [13], dietary patterns [14], and health behaviors [15]. The anxiety of the population heightened due to the uncertain prognosis of COVID-19, the imposition of unfamiliar public health measures [16], severe shortages of medicine and food [17], the loss of finances [18], and conflicting messages from authorities [19]. Those undergoing quarantine experienced stress, irritability, panic, depression, insomnia, fear, confusion, anger, frustration, boredom, and stigmatism [20,21,22,23,24]. Inadvertently, health systems prioritize screenings and control of disease transmissions ahead of managing the mental health and well-being of the population [25,26,27].

The interplay of physical, social, and economic factors during the pandemic adversely affected the mental health of previously healthy people and exacerbated mental conditions for those with pre-existing disorders [28,29]. Phobic anxiety, panic buying, doom scrolling, travelling against movement restriction orders, absconding from treatment facilities, and binge-watching were associated with impairment of self-control, mental exhaustion, sleep, and mood disturbances [30,31,32,33]. Recent studies reported increased addictive disorders during the COVID-19 quarantine, such as internet addiction, online gambling, pornography, alcoholism, or drug misuse among the general population [34,35,36]. Home isolation restricted family members to their residences, aggravated household conflicts, and increased domestic violence and child maltreatment [37,38,39,40]. Meanwhile, survivors of COVID-19 experienced post-traumatic stress disorder (PTSD) with disproportionately elevated symptoms among those requiring inpatient admission, ventilation support, and treatment for pre-existing mental disorders [41,42].

Although movement control orders were necessary to curb the transmission of COVID-19, their prolonged and repetitive impositions were detrimental. These hostile experiences caused the country to endure financial stress [43,44], social disorders [45], and emotional disorders [46], which inevitably spiked cases of suicide attempts and depression [47]. Notwithstanding the severe mental impacts on Malaysians, studies have remained limited to healthcare professionals and university students, thus, neglecting the true implications of COVID-19 on the entire population [48,49,50]. With that, this study aims to determine the interplay of associations between COVID-19 knowledge, precautionary measures, mental health, and quality of life among Malaysians. It is hypothesized that the COVID-19 pandemic has caused negative impacts on mental health as well as quality of life among Malaysians. These findings are pertinent for the timely intervention of dysfunctional processes and maladaptive lifestyles that may result in the onset of psychiatric conditions [51].

## 2. Materials and Methods

### 2.1. Study Design

This cross-sectional study was conducted from 1 January 2021 to 31 December 2021. The study was conducted in full compliance with the principles outlined in the Declaration of Helsinki and Malaysia’s Good Clinical Practice [52]. Participant recruitment was done via convenient sampling, and the survey was conducted online using Google Forms. The inclusion criteria were: (1) being aged 18 and above; (2) residing in Malaysia for more than 12 months; and (3) being willing to give informed consent. On the other hand, exclusion criteria were: (1) underlying mental illness; (2) active infection with COVID-19; and (3) healthcare workers. The eligibility of each participant was confirmed according to the protocol checklist, and their written informed consent was obtained. The study was approved by the principal investigator’s institutional ethics committee (UCSI University, Malaysia, approval code IEC-2020-FMHS-046).

### 2.2. Knowledge about COVID-19

A validated questionnaire developed by Zhong and colleagues was modified slightly for use in assessing participants’ understanding of COVID-19 [53]. The questionnaire consisted of twelve questions: four on clinical presentations, three on transmission routes, and five on prevention and control. These questions were provided with three options, namely “Yes”, “No”, and “I don’t know”. A correct answer was given 1 point, and an incorrect/not knowing answer was given 0 points. Total knowledge scores ranged from 0 to 12, with 0 to 4 points denoting low levels of knowledge, 5 to 8 points denoting moderate levels of knowledge, and 9 to 12 points denoting high levels of knowledge. The questionnaire was validated by the National Health Commission of the People’s Republic of China, indicating acceptable reliability with a Cronbach’s alpha coefficient of 0.71 [53].

### 2.3. Precautionary Behaviors

A modified version of the validated questionnaire developed by Leung and his colleagues assessed participants’ precautionary behaviors [54]. The original questionnaire was designed to assess the overall well-being and practices during SARS outbreaks in Hong Kong. In this study, only the precautionary measures section, which consists of seven questions, was used.

### 2.4. Depression, Anxiety and Stress Scales (DASS)

The validated Depression, Anxiety, and Stress Scales (DASS) were used to assess self-perceived mental distress [55]. DASS-21 is a self-report questionnaire that contains twenty-one questions, seven per subscale of depression, anxiety, and stress. Participants rated each question on a scale of 0 (did not apply to me at all) to 3 (applied to me very much or most of the time). Sum scores were computed by summing up scores within the same subscale and multiplying them by a factor of 2. The cut-off scores for the depression, anxiety, and stress subscales were 21, 15, and 26, respectively; thus, scores above these denoted high severity of mental distress [56]. DASS was previously validated for Malaysians with a Cronbach’s alpha coefficient of at least 0.74, indicating acceptable reliability [57].

### 2.5. World Health Organization Quality of Life—Brief Version (WHOQOL-BREF)

The validated World Health Organization Quality of Life—Brief Version (WHOQOL-BREF) was adopted to assess the quality of life amid the COVID-19 pandemic [58]. A WHOQOL-BREF assessment is a short-form questionnaire that determines the meaning of different aspects of life to the participants and their satisfaction with their experiences concerning those aspects of life. It is a self-perceived questionnaire consisting of four domains, namely physical health (seven items), psychological status (six items), social relationships (three items), and environmental conditions (eight items). Participants were asked to rate all the items on a Likert scale of 1 to 5 (1 = very poor, 2 = poor, 3 = neither poor nor good, 4 = good, and 5 = very good; 1 = very dissatisfied, 2 = dissatisfied, 3 = neither satisfied nor dissatisfied, 4 = satisfied, and 5 = very satisfied; 1 = not at all, 2 = a little, 3 = a moderate amount, 4 = very much, and 5 = an extreme amount; 1 = not at all, 2 = a little, 3 = moderately, 4 = mostly, and 5 = completely; 1 = not at all, 2 = a little, 3 = a moderate amount, 4 = very much, and 5 = extremely; or 1 = never, 2 = seldom, 3 = quite often, 4 = very often, and 5 = always). Items with negative scoring were reversed when summing up the total domain score. After that, it was converted to a transformed score within the range of 4 to 20. Domain scores were scaled positively, with a higher score denoting better QoL. WHOQOL-BREF was previously validated for Malaysians with a Cronbach’s alpha coefficient of 0.88, indicating good reliability [59].

### 2.6. Statistical Analysis

Categorical data were expressed in frequency and percentage, while continuous data were presented as the mean ± SD for normally distributed data or the median (interquartile range) for non-normally distributed data. Where appropriate, the association relationship was analyzed using an independent samples t-test, one-way analysis of variance (ANOVA), or a Chi-square test. Correlation analyses (Pearson’s) were performed to assess the predicted relationships between demographic, DASS, and WHOQOL-BREF outcome measures. Pearson coefficients) range from +1 to −1, with +1 representing a positive correlation, −1 representing a negative correlation, and 0 representing no relationship. Results are considered significant if *p* < 0.05. Statistical analysis was performed using SPSS 26.0 (IBM Corp., New York, NY, USA) for macOS.

## 3. Results

### 3.1. Characteristics of Participants

Of the 1246 participants who enrolled in this study, the majority (n = 506, 40.6%) were below or at the age of 30 at the time of study entry. Female participants (n = 675, 54.2%) were slightly more numerous than their male counterparts. The highest educational levels were at the pre-university and graduate levels, with 32.7% and 35.0%, respectively. Annual incomes observed a normal distribution, with the majority (n = 629, 50.5%) earning USD 10,000 to USD 20,000 in a year. Financial struggles were similar between groups. Most participants (n = 350, 28.1%) do not have any dependents living with them, followed by having two dependents (n = 279, 22.4%), and lastly having three, one, and more than three dependents with 18.9%, 16.3%, and 14.3%, respectively. Meanwhile, some participants (n = 150, or 12.0%) suffered from chronic diseases. A history of being positive for COVID-19 or being a close contact was similar between groups. The factors analyzed are normally distributed, with no significant difference between categorical variables except employment status and chronic diseases (Table 1).

### 3.2. Level of Knowledge, Precautionary Behavior, Depression, Anxiety, and Stress Scales (DASS), and Quality of Life (WHOQOL-BREF) of Participants

Most participants (n = 1097, 88.0%) showed a high level of knowledge about infectious diseases, and none had a low level of knowledge. Precautionary measures were similar for nearly all assessed behaviors, except for face mask-wearing, which was practiced by 81.5% of participants. The means (SD) of depression, anxiety, and stress were 13.7 (8.9), 13.0 (8.6), and 14.6 (8.5), respectively. With regards to severity, 69.7% had depressive symptomatology (13.1% were severe and 7.9% were extremely severe), 72.6% had anxiety symptoms (11.5% were severe and 24.3% were extremely severe), and 42.6% had stress symptoms (11.4% were wsevere and 1.4ere % extremely severe). Meanwhile, the means (SD) of physical health and psychological status were 13.0 (2.6) and 12.9 (2.6), respectively, and those for social relationships and environmental conditions were 13.5 (3.2) and 13.4 (2.4), respectively (Table 2).

### 3.3. Analysis of Association

Age, educational level, employment status, and annual incomes were found to be significantly (*p* < 0.05) associated with all DASS symptoms and QOL domains, with higher impacts on the groups of 31 to 40 years old and 41 to 50 years old (similarly high), secondary educational level, part-timer, and annual income group of less than USD 10,000. Gender was significantly (*p* < 0.05) associated with depression, anxiety, social relationships, and environmental conditions, which impacted male participants mainly. Financial struggle was significantly (*p* < 0.05) associated with anxiety and all QOL domains. Participants with one dependent were also significantly (*p* < 0.05) associated with all DASS symptoms and QOL domains, except for the environmental condition domain. A history of chronic diseases was significantly (*p* < 0.05) associated with depression, anxiety, and social relationships. In contrast, the history of being positive for COVID-19 positive or being a close contact was significantly (*p* < 0.05) associated with anxiety and stress. In addition, results indicated that participants with a moderate level of knowledge were significantly (*p* < 0.05) more impacted in terms of stress, physical health, and environmental conditions (Table 3).

### 3.4. Correlation of Coefficients

Table 4 shows the Pearson correlation coefficient matrix of the observed variables. Age was inversely correlated with knowledge (r = −0.070, *p* < 0.05), depression (r = −0.116, *p* < 0.001), anxiety (r = −0.083, *p* <0.01), and stress (r = −0.081, *p* < 0.01), and directly correlated with physical health (r = 0.102, *p* < 0.001), psychological status (r = 0.089, *p* < 0.01), social relationships (r = 0.068, *p* < 0.01), and environmental conditions (r = 0.063, *p* < 0.05). The level of knowledge was found to significantly correlate (*p* < 0.05) with anxiety (r = 0.064) directly and environmental conditions (r = −0.073) inversely.

Depression showed a strong direct correlation with anxiety (r = 0.756, *p* < 0.001) and stress (r = 0.748, *p* < 0.001), and a moderate inverse correlation with physical health (r = −0.505, *p* < 0.001), psychological status (r = −0.493, *p* < 0.001), social relationships (r = −0.431, *p* < 0.001), and environmental conditions (r = −0.419, *p* < 0.001). Anxiety showed a strong direct correlation with stress (r = 0.740, *p* < 0.001) and a moderate inverse correlation with physical health (r = −0.471, *p* < 0.001), psychological status (r = −0.438, *p* < 0.001), social relationships (r = −0.405, *p* < 0.001), and environmental conditions (r = −0.459, *p* < 0.001). Stress showed a moderate inverse correlation with physical health (r = −0.476, *p* < 0.001), psychological status (r = −0.475, *p* < 0.001), social relationships (r = −0.409, *p* < 0.001), and environmental conditions (r = −0.438, *p* < 0.001).

Meanwhile, the physical health domain of WHOQOL-BREF showed a moderate direct correlation with psychological status (r = 0.568, *p* < 0.001), social relationships (r = 0.409, *p* < 0.001), and environmental conditions (r = 0.557, *p* < 0.001). Psychological status showed a moderate direct correlation with social relationships (r = 0.403, *p* < 0.001) and environmental conditions (r = 0.524, *p* < 0.001). Lastly, social relationships were moderately and directly correlated with environmental conditions (r = 0.489, *p* < 0.001).

## 4. Discussion

The widespread morbidity and mortality associated with the COVID-19 pandemic have profoundly affected every individual’s life since the declaration of the novel coronavirus disease 2019 as an international public health emergency in January 2020 [60]. In order to limit the spread of COVID-19 and curb the drastic increase in mortality, the World Health Organization [4] recommended the implementation of Public Health and Social Measures (PHSM), such as imposing lockdown by country, state, or district on a global scale [61]. Pursuant to that, Malaysia has declared two total nationwide lockdowns during the long pandemic [62]. Prolonged lockdowns have caused inevitable changes to the usual activities, livelihoods, and routines of people, eventually leading to deteriorated mental health and increased self-harm or suicidal behavior [63]. Recent studies have pointed out that self-isolation, quarantine, spatial distancing, misleading social media content, and social and economic discord are major contributing factors to anxiety, stress, helplessness, loneliness, and depression [64,65]. Quality of life was simultaneously impacted in the general population and in post-COVID-19 patients [66,67]. Malaysia was ranked fourth in the Event Scale-Revised (IES-R) and Depression, Anxiety, and Stress Scale (DASS-21) to be impacted by the COVID-19 pandemic among seven middle-income countries in Asia [68].

The knowledge level of participants about COVID-19 was assessed using a questionnaire developed during the first outbreak in Wuhan, China [53]. The questionnaire was adopted in this study with further grouping into low, moderate, and high levels of knowledge. Results revealed that most participants (n = 1097, 88.0%) possessed a high level of knowledge after approximately two years of battling COVID-19. This finding is supported by a recent study that highlighted the direct proportional relationship between time of media exposure and perceived knowledge among the general public [69]. Prolonged lockdown periods in Malaysia have led to high dependency on various online sources to acquire updated information about the pandemic [70]. Notwithstanding the high level of COVID-19 knowledge among our participants in this study, only half were practicing precautionary measures such as covering their mouth when coughing or sneezing, using serving utensils, practicing good hygiene, or social distancing. These lax precautionary measures could be attributed to the central government’s lack of firm, persistent, and consistent enforcement. Although social distancing was strongly imposed during the beginning of the pandemic, it lacked endurance and was promptly eased following the decline in COVID-19 positive cases, increased occupancy in intensive care units (ICU), and decreased R_0_ value. Eventually, the public will lose sight of the need for social distancing and preventive measures. Similar observations were reported in India following its first wave of COVID-19 cases [71]. Second, high mask-wearing compliance could reduce adherence to social distancing, as indicated by our results. This observation can be attributed to a mechanism termed risk compensation behavior, in which individuals embrace higher risk when their safety is presumed [72].

Our results indicated the participants’ average scores for depression, anxiety, and stress to be 13.7, 13.0, and 14.6, respectively; these values were higher compared to the data reported in the most recent study [68]. The sudden hike in DASS scoring is most likely due to the prolonged lockdown implemented in 2021. Quarantine and isolation at extended lengths were deemed highly effective countermeasures for the transmission of COVID-19, but they inevitably impacted individuals’ mental health, especially their emotional well-being [73]. Growing evidence supports the negative impacts of quarantine in causing psychological distress in the form of anxiety, depression, worry, anger, confusion, and post-traumatic stress symptoms [47,73,74]. Apart from the long lockdown period, our data illustrated the potential attributions by age, gender, educational level, employment status, annual income, number of dependents, medical background of chronic illnesses, and history of being COVID-19 positive or in close contact. This is consistent with the previous findings reported in Asian countries [68,75,76,77]. Although individuals of older age (60+ years) were thought to have a greater risk of contracting and dying from COVID-19, a study has shown that this group possessed better emotional well-being, which acted as a buffer against the negative psychological impacts of COVID-19 [78]. Contrastingly, individuals younger than 50 were reported to have a more evident association with adverse mental health. This suggested that stress arising from financial insecurity is an essential risk factor for psychological morbidity, especially for those working adults between 31 and 50 years old, as observed in our study [79,80]. The faltering economy and reduction in business activities during the pandemic had a detrimental effect on workers with low income and unstable employment statuses [75]. A recent model suggested that unemployment caused by the pandemic could result in an additional 9570 suicides per year worldwide [81].

Quality of life was defined as an individual’s perception of their life status in the context of the culture and value system in which they live and concerning their goals, expectations, standards, and concerns [82]. WHOQOL was employed as a multidimensional tool to assess QoL in different aspects of life and was validated to be a useful assessment tool even in different cultural populations [83]. The average scores were 13.0, 12.9, 13.5, and 13.4 for the respective physical, psychological, social, and environmental domains. Although this scale has no cut-off score, our reported values were generally lower than previous studies focusing on specific groups (students, healthcare workers) or a specific timeframe (the first lockdown) during the beginning of the pandemic in Malaysia [84,85,86]. The predictors of QoL were age, educational level, employment status, annual income, and financial struggles for all four domains. Meanwhile, gender was accountable for social and environmental domains, and chronic disease was for social domains. Like mental health, older age appeared to be a protective factor, even though the elderly were classified as a high-risk population during the pandemic. This could be attributed to their financial stability [87], optimism, or reduced fear of death [88]. Our findings were in line with previous studies reporting older age to exhibit a similar or even better well-being status than before the pandemic [87,89,90,91,92]. As highlighted in the earlier study, older people may have better psychological strengths acquired from their life-challenging experiences, equipping them with skills to deal with adversity [93]. Apart from age and financial stability, chronic diseases were also reported to be one significant variable in determining QoL [94]. Some studies have shown that QoL is lower among patients with specific chronic non-communicable diseases (NCD) such as diabetes, hypertension, and cardiovascular disease [95,96]. Due to the fear of COVID-19 infection, populations with chronic diseases often refrain from social interactions, thus lowering their QoL in the social domain [97].

Correlation analysis revealed that age correlated negatively with knowledge, depression, anxiety, and stress, while it correlated positively with all four domains of WHOQOL. This is consistent with our speculation that information about COVID-19 was mainly acquired through social media. Older people were particularly hesitant to utilize digital services due to their refusal to learn new technologies [98]. The digital competency gap between younger and older adults is reasonably large, especially in developing countries [99]. Nonetheless, minimizing the use of social media in acquiring COVID-19 information is beneficial for reducing the symptoms of depression and anxiety [100,101]. The unverified and contradictory information on social media often caused more confusion than consolidating a consistent effort against the pandemic [101]. This study also explained the negative correlation between age and mental distress. The better QoL presented in the older population could potentially be attributed to their greater tolerance to COVID-19 risk, better sleep quality, higher optimism, and better relaxation during the pandemic [102]. The traits of depression, anxiety, and stress showed moderate negative correlations with all four domains of WHOQOL in this study. These findings concurred with previous studies reporting mental distress as a useful predictor for QoL outcomes during the pandemic [103,104,105,106]. One study highlighted that anxiety could be useful to encourage the practice of precautionary measures, but it may disrupt daily work and family life if improperly managed.

Although the pandemic is ending, previous frequent and prolonged lockdowns have caused inevitable changes for everyone. This present study indicated that prolonged lockdowns had profoundly impacted the mental health of the general population in Malaysia, reducing their quality of life during the pandemic. Employment status, financial instability, and low annual incomes appeared to be the risk factors contributing to mental distress, while older age played a protective role in contrast. To our best knowledge, this is the first large-scale study in Malaysia to assess the mental health and quality of life of the public during the pandemic. Our findings shed light on the impact of lockdowns and pandemics in the long run. Preventive measures or intervention programs such as community mental health support programs, awareness and educational campaigns, or suicide prevention programs should be implemented soonest to prevent the exacerbation of pre-existing mental conditions due to the pandemic. The primary limitation of this study is its inability to establish temporal links between outcomes and factors; the base rates of mental health symptoms compared to other time points cannot be inferred through a cross-sectional study. A longitudinal study is recommended to determine long-term mental implications involving all potential risk factors highlighted in this study.

## Figures and Tables

**Table 1:** Characteristics of Participants.

Characteristic	Frequency (%)
Age (years)	
≤30	506 (40.6)
31–40	326 (26.2)
41–50	254 (20.4)
51–60	118 (9.5)
>60	42 (3.4)
Sex	
Male	571 (45.8)
Female	675 (54.2)
Educational Level	
Secondary School	302 (24.2)
Pre-University	408 (32.7)
Graduate	436 (35.0)
Postgraduate	100 (8.0)
Employment Status	
Full-time	1012 (81.2)
Part-time	101 (8.1)
Retired	38 (3.0)
Unemployed	95 (7.6)
Annual Incomes (USD)	
Less than USD 10,000	477 (38.3)
USD 10,000 to USD 20,000	629 (50.5)
More than USD 20,000	140 (11.2)
Financial Struggle	
Yes	605 (48.6)
No	641 (51.4)
Number of Dependents	
None	350 (28.1)
1	203 (16.3)
2	279 (22.4)
3	235 (18.9)
More than 3	179 (14.3)
Chronic Diseases	
Yes	150 (12.0)
No	1096 (88.0)
History of COVID-19 Positive/Close Contact	
Yes	601 (48.2)
No	645 (51.8)

**Table 2:** Knowledge Level, Precautionary Behavior, Depression, Anxiety, and Stress Scales (DASS), and Quality of Life (WHOQOL-BREF) of Participants.

Characteristic	Frequency (%)	Mean (SD)
Knowledge Level		
Moderate	149 (12.0)	
High	1097 (88.0)	
Precautionary Behavior		
Cover mouth when coughing or sneezing	639 (51.3)	
Use serving utensils	582 (46.7)	
Wash hands with soap	645 (51.8)	
Wash hands immediately after sneezing, coughing, or rubbing nose	632 (50.7)	
Wear face mask	1015 (81.5)	
Practice social distancing	633 (50.8)	
Wash hands after touching possible contaminated objects	635 (51.0)	
DASS		
Depression		13.7 (8.9)
Anxiety		13.0 (8.6)
Stress		14.6 (8.5)
WHOQOL-BREF		
Physical health		13.0 (2.6)
Psychological status		12.9 (2.6)
Social relationships		13.5 (3.2)
Environmental conditions		13.4 (2.4)

**Table 3:** ANOVA Analysis of the Association of Depression, Anxiety, and Stress Scale (DASS) and Quality of Life (WHOQOL-BREF) with Sociodemographic and Level of Knowledge among Participants.

Characteristic	DASS (*p*-Value)			WHOQOL-BREF (*p*-Value)			
	Depression	Anxiety	Stress	Physical Health	Psychological Status	Social Relationships	Environmental Conditions
Sociodemographic Age	<0.001 ***	<0.001 ***	<0.001 ***	<0.001 ***	<0.001 ***	<0.001 ***	<0.001 ***
Sex	<0.01 **	<0.05 *	0.74	0.211	0.240	<0.01 **	<0.01 **
Educational level	<0.001 ***	<0.001 ***	<0.001 ***	<0.001 ***	<0.001 ***	<0.001 ***	<0.001 ***
Employment status	<0.001 ***	<0.001 ***	<0.001 ***	<0.001 ***	<0.001 ***	<0.001 ***	<0.001 ***
Annual incomes	<0.001 ***	<0.001 ***	<0.001 ***	<0.001 ***	<0.001 ***	<0.001 ***	<0.001 ***
Financial Struggle	0.105	<0.05 *	0.066	<0.001 ***	<0.001 ***	<0.001 ***	<0.001 ***
Number of dependents	<0.001 ***	<0.001 ***	<0.001 ***	<0.05 *	<0.01 **	<0.01 **	0.079
Chronic diseases	<0.05 *	<0.01 **	0.103	0.166	0.346	<0.05 *	0.243
History of COVID-19 positive/close contact	0.208	<0.01 **	<0.05 *	0.248	0.469	0.371	0.212
Level of Knowledge	0.061	0.104	<0.05 *	<0.01 **	0.468	0.404	<0.01 **

* Association is significant at the 0.05 level. ** Association is significant at the 0.01 level. *** Association is significant at the 0.001 level.

**Table 4:** Pearson Correlation Coefficient Matrix of the Measured Variables.

Variables	1.	2.	3.	4.	5.	6.	7.	8.	9.
Age	1								
2. Knowledge	−0.070 *	1							
3. Depression	−0.116 ***	0.045	1						
4. Anxiety	−0.083 **	0.064 *	0.756 ***	1					
5. Stress	−0.081 **	0.048	0.748 ***	0.740 ***	1				
6. Physical Health	0.102 ***	0.051	−0.505 ***	−0.471 ***	−0.476 ***	1			
7. Psychological Status	0.089 **	−0.004	−0.493 ***	−0.438 ***	−0.475 ***	0.568 ***	1		
8. Social Relationships	0.068 **	−0.015	−0.431 ***	−0.405 ***	−0.409 ***	0.409 ***	0.403 ***	1	
9. Environmental Conditions	0.063 *	−0.073 *	−0.419 ***	−0.459 ***	−0.438 ***	0.557 ***	0.524 ***	0.489 ***	1

* Correlation is significant at the 0.05 level. ** Correlation is significant at the 0.01 level. *** Correlation is significant at the 0.001 level.

## Data Availability

Not applicable.
